# Ethanolic Extracts of *Pluchea indica* Induce Apoptosis and Antiproliferation Effects in Human Nasopharyngeal Carcinoma Cells

**DOI:** 10.3390/molecules200611508

**Published:** 2015-06-22

**Authors:** Chiu-Li Kao, Joshua Cho, Ya-Zhe Lee, Yuan-Bin Cheng, Chih-Yen Chien, Chung-Feng Hwang, Yi-Ren Hong, Chao-Neng Tseng, Chung-Lung Cho

**Affiliations:** 1 Department of Biological Sciences, National Sun Yat-sen University, Kaohsiung 80424, Taiwan; E-Mails: joe7day@yahoo.com.tw (C.-L.K); m992010002@gmail.com (Y.-Z.L); 2 Department of Nursing, Tzu Hui Institute of Technology, Ping-Tung County 92641, Taiwan; 3 Department of Biology, University of Rochester, Rochester, NY 14627, USA; E-Mail: jcho23@u.rochester.edu; 4 Graduate Institute of Natural Products, School of Pharmacy, Kaohsiung Medical University, Kaohsiung 80708, Taiwan; E-Mail: jmb@kmu.edu.tw; 5 Department of Otolaryngology, Kaohsiung Chang Gung Memorial Hospital and Chung Gung University College of Medicine, Kaohsiung 83301, Taiwan; E-Mails: cychien3965@adm.cgmh.org.tw (C.-Y.C.); cfhwang.tw@gmail.com (C.-F.H.); 6 Department of Biochemistry, Faculty of Medicine, Kaohsiung Medical University, Kaohsiung 80708, Taiwan; E-Mail: m835016@cc.kmu.edu.tw

**Keywords:** *Pluchea indica*, nasopharyngeal carcinoma (NPC), p53, apoptosis

## Abstract

*Pluchea indica* is used in traditional medicine for the treatment of lumbago, ulcer, tuberculosis and inflammation. The anti-cancer activities and the underlying molecular mechanisms of the ethanolic extracts of *P. indica* root (PIRE) were characterized in the present study. PIRE strongly inhibited the viability of the human nasopharyngeal carcinoma cells (NPC-TW 01 and NPC-TW 04) in a time- and dose-dependent manner. Migration of cancer cells was also suppressed by PIRE. In addition, PIRE significantly increased the occurrence of the cells in sub-G1 phase and the extent of DNA fragmentation in a dose-dependent manner, which indicates that PIRE significantly increased apoptosis in NPC cells. The apoptotic process triggered by PIRE involved up-regulation of pro-apoptotic Bax protein and down-regulation of anti-apoptotic Bcl-2 protein, consequently increasing the ratios of Bax/Bcl-2 protein levels. Moreover, the p53 protein was up-regulated by PIRE in a concentration-dependent manner. Therefore, PIRE could induce the apoptosis-signaling pathway in NPC cells by activation of p53 and by regulation of apoptosis-related proteins.

## 1. Introduction

While nasopharyngeal carcinoma (NPC) is an uncommon form of head and neck cancer in most parts of the world [1], a high incidence is found among the Inuit of Alaska, native Greenlanders and the southern Chinese population of Guangdong [2,3]. Epstein-Barr virus infection plays important roles in the pathogenesis of NPC [4]. NPC detection in the early stage is often difficult because the symptoms are non-specific; thus, the majority of NPC is diagnosed at an advanced stage [5]. The tumor-node-metastasis (TNM) staging system for NPC is the main evaluation of prognosis [6]. NPC is highly sensitive to radiation; thus, a common choice of early-stage NPC treatment is radiotherapy. However, after single-modality treatment with radiotherapy, approximately 70% patients newly diagnosed with TNM stage III or IV NPC are still afflicted with local recurrence or distant metastases [7]. Therefore, a combined chemo-radiation therapy is routinely used to improve the survival rates of patients with advanced NPC [8]. Nevertheless, even with a combined chemo-radiation treatment, survival rates for patients with metastatic NPC remain poor, with a very high recurrence rate of up to 82% [9]. Therefore, it is important to develop new drugs that are effective for the treatment of NPC. Numerous medicinal plants have become promising sources for the development of new drugs [10]. *Pluchea indica* (L.) Less is a well-known medicinal plant that grows naturally in litoral areas of tropical regions. It could be found in countries such as India, Myanmar, China, Philippines, Malaysia and Australia. It has been traditionally used as an astringent and an antipyretic [11]. The methanol extracts of *P.* *indica* root were shown to be effective in the neutralization of viper and cobra venoms [12]. The methanol fraction of *P.* *indica* root [13] and ethanolic extracts of *P.* *indica* leaf exhibited significant anti-inflammatory activity [14]. *In vitro* studies indicated that methanol extract of *P.* *indica* exhibited significant antioxidant activities [15]. In animal models, the methanol extract of *P.* *indica* roots showed antiulcer activity [16]. Furthermore, *P.* *indica* aqueous extract showed antiviral activity against human immunodeficiency virus type 1 (HIV-1) [17]. However, there is no information regarding the anti-cancer effect of the ethanol extract of *P. indica* in nasopharyngeal carcinoma.

In this study, the anti-cancer effect of ethanol extracts of PIRE was investigated and the molecular mechanism of PIRE-induced cell death in two human nasopharyngeal carcinoma cells lines, NPC-TW 01 and NPC-TW 04 was explored.

## 2. Results and Discussion

### 2.1. Phytochemical Screening of PIRE

A number of studies have reported that medicinal herbs can inhibit proliferation, induce apoptosis, suppress angiogenesis, delay metastasis and enhance chemotherapy, exhibiting anti-cancer potential both *in vitro* and *in vivo* [18]. Part of the beneficial effects of herbal extracts is due to their having a wide variety of biologically active phytochemicals, including phenolics, flavonoids, carotenoids, alkaloids, nitrogen-containing compounds, as well as organosulfur compounds, all of which have been shown to suppress multiple molecular events related to carcinogenesis [19,20]. The preliminary phytochemical analysis performed on PIRE revealed abundant amounts of phenol, alkaloid, flavonoid and tannin (Table 1), suggesting these phytochemicals may bestow PIRE with fundamental anti-cancer activities.

**Table 1:** Phytochemical contents of PIRE.

Phytochemical Content	Mean ± SD
Total Flavonoid	112.36 ± 0.51 mg of CE/g of dry extract
Tannin	27.92 ± 0.81 mg of CE/g of dry extract
Total Phenol	209.21 ± 1.47 mg of GAE/g of dry extract
Alkaloid	32.24 ± 0.38 mg of AE/g of dry extract

Mean ± SD (*N* = 3); CE, catechin equivalents; GAE, gallic acid equivalents; AE, atropine equivalents.

### 2.2. PIRE Suppresses NPC Cell Proliferation

**Figure 1:**
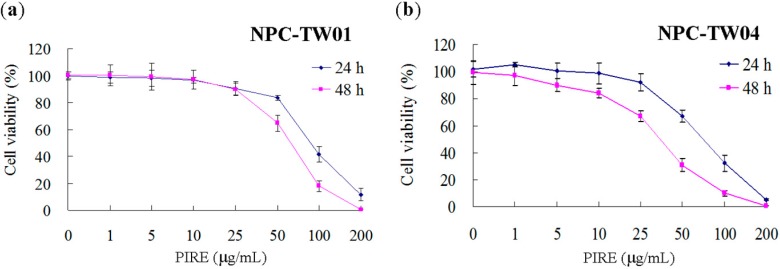
Anti-proliferative activity of PIRE in NPC-TW 01 and NPC-TW 04 cells. (**a**) NPC-TW 01 and (**b**) NPC-TW 04 cells were treated with 0–200 μg∙mL^−1^ PIRE, and viabilities were determined using WST-1 assay after 24 h and 48 h. Percent cell viability of each experimental group was calculated, with 100% representing cells treated with 0.1% DMSO alone (control). The results are the means ± SD from three experiments.

**Figure 2:**
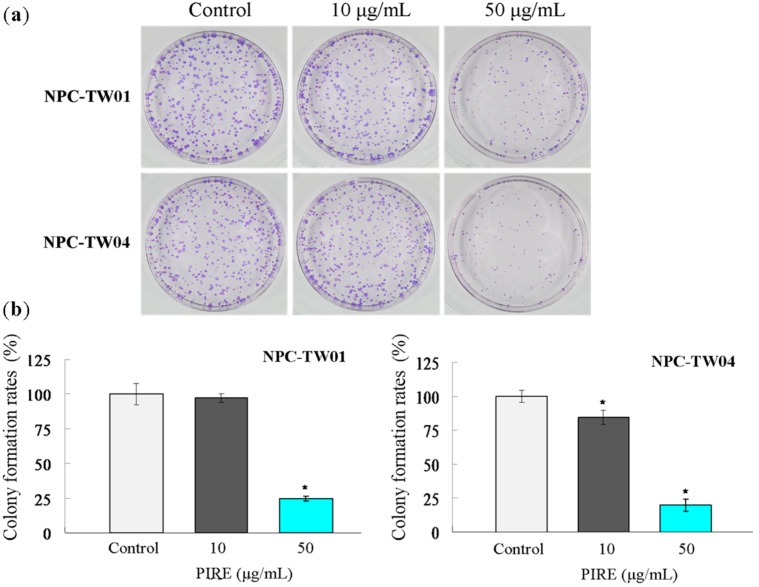
Inhibition of NPC-TW 01 and NPC-TW 04 cells colony formation by PIRE. (**a**) Cells were treated with 10 and 50 μg∙mL^−1^ PIRE or control for 24 h, and then harvested and seeded on 60-mm dishes for 10 d culture. Growth was measured by counting the colony number; (**b**) Results were averaged from three independent experiments and presented as means ± SD. * Means significantly different from control (0.1% DMSO) at the same dose at *p* < 0.05.

To investigate the possible cytotoxic effect of PIRE on NPC cells, two lines of NPC cells were treated with 0 to 200 μg∙mL^−1^ PIRE for 24 h to 48 h and the cell survival was estimated by WST-1 reagent. PIRE reduced the survival of both NPC cells in a dose-dependent manner with 100 μg∙mL^−1^ PIRE reducing cellular survival to less than 50% of control (Figure 1). It seemed that NPC-TW 04 cells are slightly more sensitive to PIRE than NPC-TW01 cells. Concentrations of the extracts that exhibited 50% growth inhibition (IC_50_ value) in NPC-TW 01 and NPC-TW 04 cells were 108.5 ± 3.09 μg∙mL^−1^ and 93.2 ± 5.88 μg∙mL^−1^, respectively at 24 h, and 83.15 ± 5.72 μg∙mL^−1^ and 63.41 ± 4.16 μg∙mL^−1^, respectively at 48 h. Further confirmation of the growth-suppressive property of PIRE was obtained using the colony formation assay. Cells were treated with 0, 10 and 50 μg∙mL^−1^ PIRE for 24 h and then seeded at clonal density for an additional 10-day culture. PIRE at 50 μg∙mL^−1^ effectively suppressed colony formation in both cell lines (Figure 2a). Colony forming efficiencies of NPC-TW 01 cells pre-treated with 10 and 50 μg∙mL^−1^ were 97.13% ± 3.28, 24.57% ± 1.81, respectively and 84.76% ± 5.29, 20.09% ± 4.46, respectively in NPC-TW 04 cells, confirming the effective inhibition effect of PIRE on cell proliferation (Figure 2b).

### 2.3. PIRE Inhibits NPC Cell Migration

**Figure 3:**
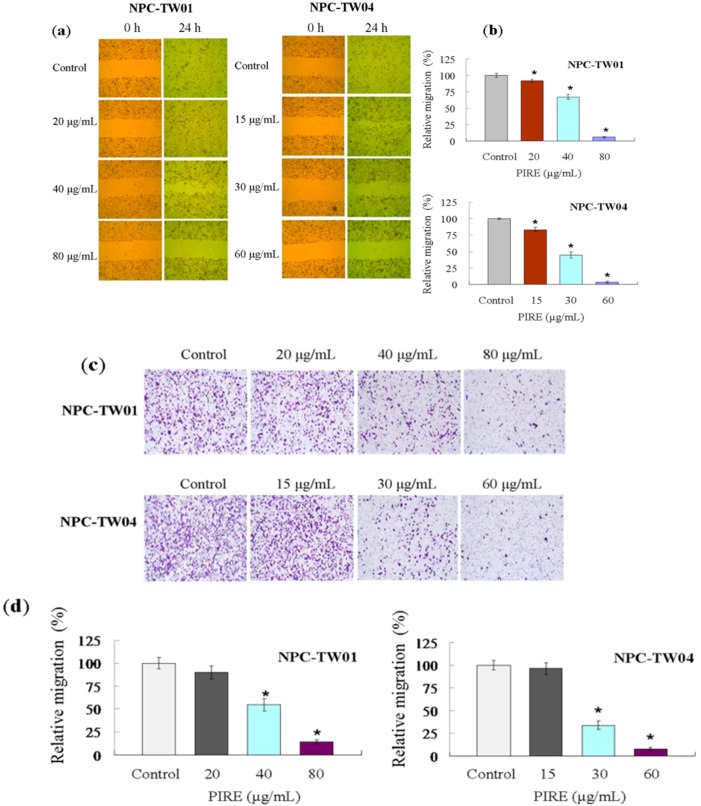
Effects of PIRE on NPC cell migration. (**a**) Images of wound of NPC-TW 01 and NPC-TW 04 cells treated with various concentration of PIRE at 0 and 24 h; (**b**) The distance between the two edges of the scratch were measured in six random fields from each treatment; (**c**) Cells migrated to the underside of the membrane in transwell migration assay were stained and quantitated; (**d**) The number of migratory cells was normalized to that of NPC cells treated with 0.1% DMSO alone (control). The results are the means ± SD from three experiments; * Means significantly different from control (0.1% DMSO) at the same dose at *p* < 0.05.

Wound healing assay was performed to examine the inhibitory effect of PIRE on the motility of NPC cells. When treated with increasing concentrations of PIRE, less migratory cells were found in the gap after 24 h of incubation (Figure 3a). The relative migration rates of NPC-TW 01 cells were 91.85%, 67.23% and 5.65% at 24 h in the presence of 20, 40 and 80 μg∙mL^−1^ PIRE, respectively. The migration rates of NPC-TW 04 cells were 83.57%, 45% and 3.47% at 24 h with 15, 30 and 60 μg∙mL^−1^ PIRE, respectively (Figure 3b). The transwell assay was used to further investigate whether the invasiveness of NPC cells was affected. As indicated in Figure 3c, the ability of NPC cells to across the membrane filter was also significantly inhibited by 30 to 80 μg∙mL^−1^ PIRE. The average percentages of migratory NPC-TW 01 cells treated with 20, 40 and 80 μg∙mL^−1^ PIRE for 24 h, compared to control, were 90%, 54.63% and 14.07%, respectively, and 96.51%, 33.78% and 7.76% in NPC-TW 04 cells treated with 15, 30 and 60 μg∙mL^−1^ PIRE, respectively (Figure 3c,d). Migration of cancer cells contributes greatly to tumor metastasis and the blocking of which is critical in successful cancer therapy. The effective suppressing of the migration of NPC cells by PIRE further increases the potential of PIRE as an anticancer agent.

### 2.4. PIRE Induces NPC Cell Apoptosis

**Figure 4:**
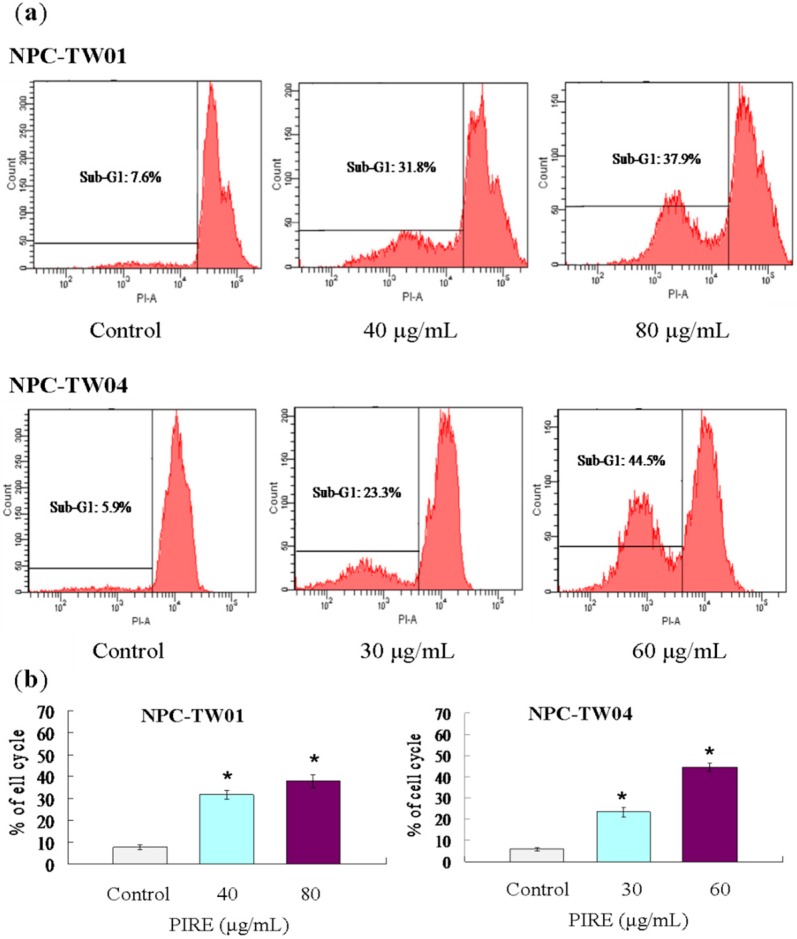
Flow cytometry analysis of NPC cells treated with PIRE. (**a**) Cells in 60 mm culture dishes were treated with varying concentrations of PIRE or 0.1% DMSO for 48 h. After incubation, both floating and adherent cells were harvested and combined for PI staining and flow cytometric analysis of subG1 population; (**b**) The percentage of subG1 determined by flow cytometric analysis. Values are means of three separate experiments; * Means significantly different from control (0.1% DMSO) at the same dose at *p* < 0.05.

**Figure 5:**
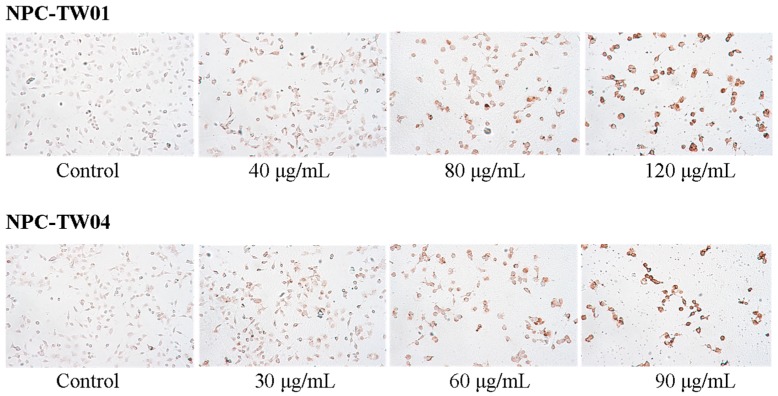
Apoptotic cells induced by PIRE in NPC cells. Detection of apoptotic cells by TUNEL assay. Cells were treated with varying concentrations of PRIE or control for 48 h. Apoptotic nuclei and fragmented DNA appeared dark brown after staining. Original magnification × 200.

To investigate the mechanism underlying the ability of PIRE to reduce NPC cells survival, we tested whether apoptosis was induced by PIRE. After 48 h PIRE treatment, cells were harvested and the sub-G1 ratio was determined by flow cytometry. The results demonstrate that treatment with 30 to 80 μg∙mL^−1^ PIRE significantly increased the cell population of sub-G1 phase (Figure 4a). When NPC-TW 01 cells were treated with 40 and 80 μg∙mL^−1^ PIRE the percentage of cells in sub-G1 phase increased by 4.2-fold and 5-fold in, respectively; and by 3.9-fold and 7.5-fold in NPC-TW 04 treated with 30 and 60 μg∙mL^−1^ PIRE, respectively (Figure 4b). NPC cells treated with PIRE were also stained by terminal deoxynucleotidyl transferase deoxyuridine 5′-triphosphate (dUTP) nick-end labeling (TUNEL) assay to detect the apoptotic cells. Increasing concentrations of PIRE decreased total cell numbers, at the same time increased TUNEL-positive NPC cell populations (Figure 5).

### 2.5. Effect of PIRE on the Expression of Apoptosis-Related Proteins

**Figure 6:**
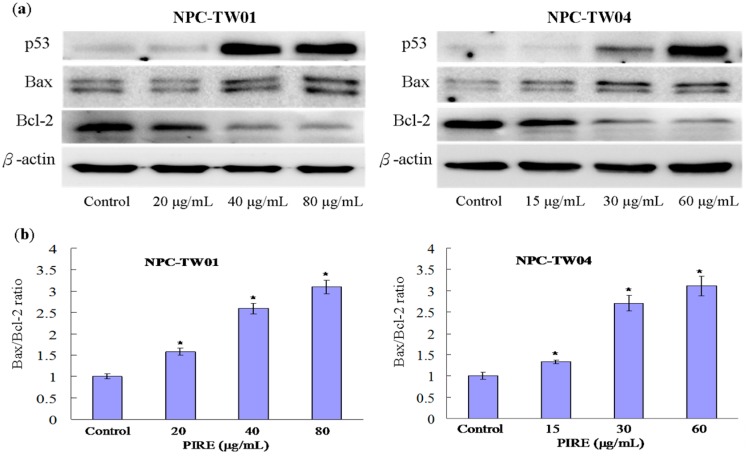
Effect of PIRE on the expression of p53, Bax and Bcl-2 proteins in NPC cells. (**a**) Cells were treated with varying concentrations of PIRE for 48 h and the expression of p53, Bax, Bcl-2 and *β*-actin was detected by Western blotting analysis; (**b**) The Bax/Bcl-2 ratios were increased by PIRE. The density of corresponding bands in (**a**) were quantitated and plotted as the ratio of Bax/Bcl-2. Results are the means ± SD from three experiments; * Means significantly different from control (0.1% DMSO) at the same dose at *p* < 0.05.

Two major signaling pathways leading to apoptosis are the extrinsic pathway mediated by the death receptor and the intrinsic pathway mediated by the mitochondria [21]. The intrinsic apoptotic pathway is regulated by Bcl-2 family which includes both pro-apoptotic regulators such as Bax, Bad, Bid, Bcl-xS, and anti-apoptotic regulators such as Bcl-2 and Bcl-xL [22]. The balance between pro- and anti-apoptotic proteins will determine either a cell stays alive or undergoes apoptosis*.* Indeed, the Bax/Bcl-2 ratio is crucial in determining survival or death following an apoptotic stimulus [23]. To assess whether PIRE incudes cell death by affecting key regulators of apoptosis, the expression of p53, Bax and Bcl-2 protein in NPC cells treated with PIRE was examined using western blotting analysis. The results indicated that PIRE increased the expression of tumor suppressor protein p53 and pro-apoptotic Bax protein, and at the same time decreased the expression of anti-apoptotic Bcl-2 protein (Figure 6a). As a result of this change of protein expression levels, the Bax/Bcl-2 ratios were increased by PIRE, therefore promoting apoptosis. In NPC-TW 01 cells, the Bax/Bcl-2 ratio increased approximately 1.58, 2.59 and 3.1-fold in the presence of 20, 40 and 80 μg∙mL^−1^ PIRE, respectively. In NPC-TW 04 cells treated with 15, 30 and 60 μg∙mL^−1^ PIRE, this ratio increased approximately 1.34, 2.71 and 3.12-fold, respectively (Figure 6b). The tumor suppressor p53 has been implicated in many key cellular processes, including DNA repair, cell cycle arrest, and apoptosis [24]. It is reported that p53 could directly activate the Bax and engage the apoptotic program, presumably due to its ability to modulate the Bax/Bcl-2 or Bcl-xL/Bax ratio [25]. Our results imply that PIRE could activate p53 and consequently affect the expression of its downstream effectors, such as the Bcl-2 family proteins.

### 2.6. Discussion

The *P.* *indica* have been used as a traditional medicine for the treatment of various diseases. The flower and leaf extracts of *P. indica* exhibited antituberculosis activity with a minimum inhibitory concentration (MIC) of 800 μg∙mL^−1^ [26]. The caffeoylquinic acid derivatives isolated from leaves of *P. indica* could prolong postprandial hyperglycaemia by inhibiting á-glucosidase, suggesting that *P. indica* has a medicinal supplement for diabetes mellitus treatment and prevention [27]. The ethyl acetate fraction of ethanol extract of *P.* *indica* leaves (EFPI) exhibited potent anti-inflammatory activity through inhibition of NO production and iNOS expression by blocking NF-kB [14]. Previous studies reported that expression of iNOS induces oxidative stress in NPC [28,29] and NF-κB pathway has been suggested to be involved in the NPC development [30,31].

Previous study has demonstrated that crude aqueous extract of *P. indica* root could suppress proliferation, viability, and migration of GBM8401 and HeLa cells [32]. In the present study, the WST-1 assays and colony formation assay showed that PIRE could inhibit proliferation of the both NPC cells in a dose-dependent manner. PIRE had a more evident cytotoxic effect on NPC-TW04 than NPC-TW01 cells. NPC has higher metastatic potential than other head and neck cancers [33]. Distant metastasis is the major cause of treatment failure. Prevention, prediction and inhibition of NPC metastasis is critical to further improve the survival rate [34,35]. Studies had shown that there were significant correlation between matrix metalloproteases (MMPs) activity and the invasion and metastasis of NPC [36,37]. Previous study reported that quinic acid esters from the leaves of *P. indica* effectively inhibited collagenase, MMP-2 and MMP-9 [38]. In current study, the data showed that PIRE were effective in suppressing the migration of NPC-TW01 and NPC-TW04 cells. Induction of apoptosis is a key cytotoxic action of antitumor agents. A number of natural compounds isolated from plants induce apoptotic pathways in cancer cells through various mechanisms [39]. Previous study showed that *P. indica* root aqueous extract at 0.5 μg∙mL^−1^ concentration effectively promoted apoptosis in HeLa cells [32]. It was found that phosphorylated-p53 and p21 were induced and phosphorylated-AKT were decreased in HeLa cells treated with *P. indica* root aqueous extracts [32]. The tumor suppressor p53 is activated in response to DNA damage and is estimated that over 50% of human tumors harbor mutations in the *TP53* gene [40,41]. It plays a key role in the regulation of G2/M and G1 cell-cycle arrest, DNA damage recognition, DNA repair, apoptosis, and senescence [42]. p53 could trigger apoptosis by initiating transcription of pro-apoptotic proteins such as PUMA, Bax, Bid, CD95, and TRAIL-R2 [43]. In addition, a fraction of p53 localizes to the mitochondria following apoptotic stimuli where it directly induces mitochondrial permeabilization and cytochrome c release by forming inhibitory complexes with the Bcl-xL and Bcl-2 [44]. Furthermore, cytosolic p53 can activate pro-apoptotic proteins such as Bax and Bak through direct protein–protein interaction [45]. In this study, PIRE induced apoptosis in NPC cells by up-regulating the protein levels of p53 and Bax, while down-regulating the levels of Bcl-2. The activation of p53 has been shown to be involved in the radioresponse in NPC [46] and injection of exogenous *TP53* gene into NPC patients significantly improved radiosensitivity [47]. Our findings warrant further studies on the molecular mechanism underlying the anti-cancer activity of *P. indica*, and on its potential therapeutic use in anti-cancer treatment.

## 3. Experimental Section

### 3.1. General Experimental Procedures

Preliminary phytochemical analysis was determined on a spectrophotometer (U-100, Hitachi, Tokyo, Japan). Flow cytometric analysis was performed by using a BD LSR II Flow Cytometer (BD Biosciences, San Jose, CA, USA). Western blots were analyzed by a CCD camera (FUJIFILM, LAS 3000, Tokyo, Japan) and densitometry was performed using Multi Gauge V2.02 (FUJIFILM).

### 3.2. Plant Material

The roots of *P. indica* were collected from the Yanchiao campus of National Kaohsiung Normal University, Kaohsiung, Taiwan, in August, 2013. The plant material was authenticated by Dr. L.-J. Liao. The samples were deposited at Sun Yat-sen University, Kaohsiung, Taiwan.

### 3.3. Extraction and Isolation

The dried powder of the roots of *P.* *indica* (309.9 g) was extracted three times with 95% (v/v) ethanol at room temperature overnight and filtered. The ethanol extracts was partitioned between water and ethyl acetate (v/v 1:1). The ethyl acetate fraction was partitioned in 1:1 (v/v) mixture of 75% ethanol and hexanes. The 75% ethanol layer was concentrated to give PIRE (2.6 g). PIRE was dissolved in dimethyl sulfoxide (DMSO). The final concentration of DMSO in the culture medium was less than 0.1% (v/v). Cells grown in medium containing 0.1% DMSO without PIRE served as control.

### 3.4. Preliminary Phytochemical Analysis

The concentrations of phenolic compounds and flavonoids in extracts were determined by using Folin-Ciocalteu’s reagent and the aluminum chloride colorimetric method and were calculated as gallic acid equivalents in mg∙g^−1^ of extract and catechin equivalents in mg∙g^−1^ of extract, respectively. Condensed tannins (proanthocyanidins) were determined according to the vanillin assay and expressed as catechin equivalents in mg∙g^−1^ of extract [32]. Alkaloids were measured by bromocresol green method and expressed as atropine equivalents in mg∙g^−1^ of extract [48]. All assays were carried out in triplicate.

### 3.5. Cell Culture

Two nasopharyngeal carcinoma cell lines, NPC-TW 01 and NPC-TW 04 (a gift from Dr. Chin-Tarng Lin, National Taiwan University, Taipei, Taiwan), were cultured in Dulbecco’s modified Eagle’s medium (DMEM) (Hyclone, Logan, UT, USA) with 10% fetal bovine serum (FBS) (Hyclone, Logan, UT, USA). NPC-TW 01 is a keratinizing squamous cell carcinoma line, and NPC-TW 04 is an undifferentiated carcinoma line [49]. Cells were incubated at 37 °C, in a humidified incubator with 5% CO_2_.

### 3.6. Cell Viability Assays

Cell proliferation was evaluated using the WST-1 colorimetric assay. Cells were seeded into 96-well plates at a density of 2000 cells per well. Subsequent to 24 h incubation, cells were treated with varying concentrations (0–200 μg∙mL^−1^) of PIRE or control (0.1% DMSO) for 24 and 48 h. After treatment, medium containing PIRE was carefully removed, and the cell proliferation reagent WST-1 (Roche, Mannheim, Germany) was added to each well and incubated for an additional 3 h at 37 °C. The absorption at 450 nm was then measured by an ELISA plate reader (Micro Quant, BioTek, Winooski, VT, USA), and the IC_50_ values were obtained for these cell lines, as previously described [32].

### 3.7. Colony Formation Assay

Cells were treated with varying concentrations of PIRE or control for 24 h. Thereafter, the cells were harvested and seeded on 6-cm dishes at 200 cells per dish and cultured at 37 °C, 5% CO_2_ for 10 days. The plates were prepared in triplicates. Colonies were then stained with crystal violet and counted under a dissection microscope.

### 3.8. In Vitro Wound Healing Assay

Cells were seeded in a 6-well plate at 3 × 10^4^ per well and grown to 90% confluence. The cell monolayer was scraped with a sterile micropipette tip to create gaps of constant width. After washing with PBS, cells were incubated in various concentrations of PIRE or control. Wound closure was observed and photographed at 0 and 24 h with a microscope. The wound area was quantified using ImageJ version 1.29 (National Institutes of Health, Bethesda, MD, USA) software. Wound healing was defined as the percentage of the starting distance between the two edges of each wound.

### 3.9. Migration Assay

The *in vitro* migration assays was modified from previous studies [50]. Cell migration assays were performed using 24-well cell culture inserts (8-μm pore size, Nunc, Roskilde, Denmark). Cells at a density of 1 × 10^4^ were seeded in the upper chambers with 100 μL of 0.5% FBS/DMEM containing varying concentrations of PIRE or control. The lower chamber was filled with 800 μL of DMEM with 20% FBS. After incubation for 9 h, the medium in the upper chamber was removed and the filters were fixed with 4% paraformaldehyde for 15 min. The cells on the upper surface of the filters were removed with cotton swabs and the cells on the lower surface of the filter were stained with crystal violet for 5 min, and 6 randomized fields were counted at 200 × magnification under a microscope.

### 3.10. Flow Cytometric Analysis

Determination of apoptosis was conducted by staining with propidium iodide (PI) (BD Pharmingen, BD Biosciences, San Diego, CA, USA). Cells were seeded in 6-well plates at a density of 1 × 10^5^ cells/mL and incubated with various concentrations of PIRE. After 24 h, cells were fixed with 70% ethanol at 4 °C overnight. Cells were then washed twice with PBS and resuspended in 500 μL PBS with 10 μL RNase A (Sigma, Saint Louis, MO, USA), and incubated at 37 °C for 30 min. After adding 20 μL PI and mixing at 4 °C for 3 h in the dark, flow cytometric analysis was performed.

### 3.11. TUNEL Staining

Evaluation of apoptotic cells was performed using the Apoptag *in situ* apoptosis detection kit (Chemicon International, Temecula, CA, USA) according to the manufacturer’s instruction. Cells grown on coverslips were incubated in various concentrations of PIRE or control for 48 h. The cells were fixed with 1% paraformaldehyde for 10 min at room temperature, with post-fixation in pre-cooled ethanol:acetic acid (2:1) for 5 min at −20 °C. Endogenous peroxidase activity was quenched using 3.0% hydrogen peroxide/PBS for 5 min at room temperature. Cells were incubated with terminal deoxynucleotidyl transferase (TdT) enzyme for 1 h at 37 °C followed by anti-digoxigenin peroxidase for 30 min at room temperature, and then stained with diaminobenzidine (DAB). The TUNEL-positive cells were stained dark brown. Methyl green (Merck, Darmstadt, Germany) was used as counterstain.

### 3.12. Western Blot Analysis

Cells cultured in 10-cm dishes were treated with 0–80 μg∙mL^−1^ PIRE for 48 h and then lysed with T-PER protein extraction reagent (Pierce, Rockford, IL, USA). Cell lysates (30 μg total protein) were separated by sodium dodecyl sulfate-polyacrylamide gel electrophoresis and transferred to nitrocellulose membranes and probed with the following antibodies: p53 (DAKO), Bax, Bcl-2 (Santa Cruz Biotechnology, San Jose, CA, USA), or *β*-actin (Sigma), respectively. The immunoblots were developed and visualized by the enhanced chemiluminescence detection system (Amersham ECL Plus Western Blotting Detection Reagents; GE Healthcare UK Limited., Buckinghamshire, HP7 9NA, UK). Images were scanned with LAS-3000 (FUJIFILM) and protein bands were quantitated densitometrically using Multi Gauge V2.02 (FUJIFILM). Each experiment was performed three times.

### 3.13. Statistical Analysis

Data are presented as mean ± standard deviations (SD) of three independent experiments and statistical significance was determined using independent Student’s *t*-test with SigmaPlot-Systat Software (Systat Software Inc., San Jose, CA, USA). A significant difference was considered if *p* < 0.05.

## 4. Conclusions

Although many reports have demonstrated that *P. indica* possesses important pharmacological activities; none has studied the effect on NPC. The current study showed that PIRE has strong anti-cancer activity against NPC *in vitro*. Specifically, our results showed that PIRE induces apoptosis in NPC cells by up-regulating the level of p53 and the level of Bax, while down-regulating the level of Bcl-2. Therefore, PIRE could be further investigated as an alternative chemotherapeutic agent for NPC.

## References

[1] Chang E.T., Adami H.O. (2006). The enigmatic epidemiology of nasopharyngeal carcinoma. Cancer Epidemiol. Biomarkers Prev..

[2] Parkin D.M., Muir C.S. (1992). Cancer Incidence in Five Continents. Comparability and quality of data. IARC Sci. Publ..

[3] Buell P. (1974). The effect of migration on the risk of nasopharyngeal cancer among Chinese. Cancer Res..

[4] Pegtel D.M., Subramanian A., Sheen T.S., Tsai C.H., Golub T.R., Thorley-Lawson D.A. (2005). Epstein-Barr-virus-encoded LMP2A induces primary epithelial cell migration and invasion: Possible role in nasopharyngeal carcinoma metastasis. J. Virol..

[5] Tabuchi K., Nakayama M., Nishimura B., Hayashi K., Hara A. (2011). Early detection of nasopharyngeal carcinoma. Int. J. Otorhinolaryngol..

[6] Lu J.C., Wei B.Q., Chen W.Z., Qian P.D., Zhang Y.Q., Wei Q., Cha W.W., Li F., Ni M. (2006). Staging of nasopharyngeal carcinoma investigated by magnetic resonance imaging. Radiother. Oncol..

[7] Teo P., Yu P., Lee W.Y., Leung S.F., Kwan W.H., Yu K.H., Choi P., Johnson P.J. (1996). Significant prognosticators after primary radiotherapy in 903 nondisseminated nasopharyngeal carcinoma evaluated by computer tomography. Int. J. Radiat. Oncol. Biol. Phys..

[8] Al-Sarraf M., LeBlanc M., Giri P.G., Fu K.K., Cooper J., Vuong T., Forastiere A.A., Adams G., Sakr W.A., Schuller D.E. (1998). Chemoradiotherapy versus radiotherapy in patients with advanced nasopharyngeal cancer: Phase III randomized Intergroup study 0099. J. Clin. Oncol..

[9] Cheng S.H., Tsai S.Y., Yen K.L., Jian J.J., Chu N.M., Chan K.Y., Tan T.D., Cheng J.C., Hsieh C.Y., Huang A.T. (2000). Concomitant radiotherapy and chemotherapy for early-stage nasopharyngeal carcinoma. J. Clin. Oncol..

[10] Tan W., Lu J., Huang M., Li Y., Chen M., Wu G., Gong J., Zhong Z., Xu Z., Dang Y. (2011). Anti-cancer natural products isolated from chinese medicinal herbs. Chin Med..

[11] Ab Rahman M.R., Abdul Razak F., Mohd Bakri M. (2014). Evaluation of wound closure activity of *Nigella sativa*, *Melastoma malabathricum*, *Pluchea indica*, and *Piper sarmentosum* Extracts on scratched monolayer of human gingival fibroblasts. Evid. Based Complement. Alternat. Med..

[12] Gomes A., Saha A., Chatterjee I., Chakravarty A.K. (2007). Viper and cobra venom neutralization by beta-sitosterol and stigmasterol isolated from the root extract of *Pluchea indica* Less. (Asteraceae). Phytomedicine.

[13] Sen T., Nag Chaudhuri A.K. (1991). Antiinflammatory evaluation of a *Pluchea indica* root extract. J. Ethnopharmacol..

[14] Buapool D., Mongkol N., Chantimal J., Roytrakul S., Srisook E., Srisook K. (2013). Molecular mechanism of anti-inflammatory activity of *Pluchea indica* leaves in macrophages RAW 264.7 and its action in animal models ofinflammation. J. Ethnopharmacol..

[15] Sen T., Dhara A.K., Bhattacharjee S., Pal S., Nag Chaudhuri A.K. (2002). Antioxidant activity of the methanol fraction of *Pluchea indica* root extract. Phytother. Res..

[16] Sen T., Ghosh T.K., Chaudhuri A.K. (1993). Studies on the mechanism of anti-inflammatory and anti-ulcer activity of *Pluchea indica*—probable involvement of 5-lipooxygenase pathway. Life Sci..

[17] Locher C.P., Witvrouw M., De Bethune M.P., Burch M.T., Mower H.F., Davis H., Lasure A., Pauwels R., de Clercq E., Vlietinck A.J. (1996). Antiviral activity of Hawaiian medicinal plants against human immunodeficiency Virus Type-1 (HIV-1). Phytomedicine.

[18] Park H.-R., Lee H.-S., Cho S.Y., Kim Y.-S., Shin K.-S. (2013). Anti-metastatic effect of polysaccharide isolated from *Colocasia esculenta* is exerted through immunostimulation. Int. J. Mol. Med..

[19] Karna P., Gundala S.R., Gupta M.V., Shamsi S.A., Pace R.D., Yates C., Narayan S., Aneja R. (2011). Polyphenol-rich sweet potato greens extract inhibits proliferation and induces apoptosis in prostate cancer cells *in vitro* and *in vivo*. Carcinogenesis.

[20] Nishino H., Satomi Y., Tokuda H., Masuda M. (2007). Cancer control by phytochemicals. Curr. Pharm. Des..

[21] Fulda S., Debatin K.M. (2006). Extrinsic versus intrinsic apoptosis pathways in anticancer chemotherapy. Oncogene.

[22] Elmore S. (2007). Apoptosis: A review of programmed cell death. Toxicol. Pathol..

[23] Del Poeta G., Venditti A., del Principe M.I., Maurillo L., Buccisano F., Tamburini A., Cox M.C., Franchi A., Bruno A., Mazzone C. (2003). Amount of spontaneous apoptosis detected by Bax/Bcl-2 ratio predicts outcome in acute myeloid leukemia (AML). Blood.

[24] Yu Q. (2006). Restoring p53-mediated apoptosis in cancer cells: New opportunities for cancer therapy. Drug Resist. Updat..

[25] Chipuk J.E., Kuwana T., Bouchier-Hayes L., Droin N.M., Newmeyer D.D., Schuler M., Green D.R. (2004). Direct activation of Bax by p53 mediates mitochondrial membrane permeabilization and apoptosis. Science.

[26] Mohamad S., Zin N.M., Wahab H.A., Ibrahim P., Sulaiman S.F., Zahariluddin A.S., Noor S.S. (2011). Antituberculosis potential of some ethnobotanically selected Malaysian plants. J. Ethnopharmacol..

[27] Arsiningtyas I.S., Gunawan-Puteri M.D., Kato E., Kawabata J. (2014). Identification of alpha-glucosidase inhibitors from the leaves of *Pluchea indica* (L.) Less., a traditional Indonesian herb: Promotion of natural product use. Nat. Prod. Res..

[28] Segawa Y., Oda Y., Yamamoto H., Uryu H., Shiratsuchi H., Hirakawa N., Tomita K., Yamamoto T., Oda S., Yamada T. (2008). Overexpression of inducible nitric oxide synthase and accumulation of 8-OHdG in nasopharyngeal carcinoma. Histopathology.

[29] Murata M., Thanan R., Ma N., Kawanishi S. (2012). Role of nitrative and oxidative DNA damage in inflammation-related carcinogenesis. J. Biomed. Biotechnol..

[30] Ren Q., Sato H., Murono S., Furukawa M., Yoshizaki T. (2004). Epstein-Barr virus (EBV) latent membrane protein 1 induces interleukin-8 through the nuclear factor-kappa B signaling pathway in EBV-infected nasopharyngeal carcinoma cell line. Laryngoscope.

[31] Sun W., Guo M.-M., Han P., Lin J.-Z., Liang F.-Y., Tan G.-M., Li H., Zeng M., Huang X. (2012). Id-1 and the p65 subunit of NF-kappaB promote migration of nasopharyngeal carcinoma cells and are correlated with poor prognosis. Carcinogenesis.

[32] Cho J.J., Cho C.L., Kao C.L., Chen C.M., Tseng C.N., Lee Y.Z., Liao L.J., Hong Y.R. (2012). Crude aqueous extracts of *Pluchea indica* (L.) Less. inhibit proliferation and migration of cancer cells through induction of p53-dependent cell death. BMC Complement. Altern. Med..

[33] Ahmad A., Stefani S. (1986). Distant metastases of nasopharyngeal carcinoma: A study of 256 male patients. J. Surg. Oncol..

[34] Li X.-J., Peng L.-X., Shao J.-Y., Lu W.-H., Zhang J.-X., Chen S., Chen Z.-Y., Xiang Y.-Q., Bao Y.-N., Zheng F.-J. (2012). As an independent unfavorable prognostic factor, IL-8 promotes metastasis of nasopharyngeal carcinoma through induction of epithelial-mesenchymal transition and activation of AKT signaling. Carcinogenesis.

[35] Geara F.B., Sanguineti G., Tucker S.L., Garden A.S., Ang K.K., Morrison W.H., Peters L.J. (1997). Carcinoma of the nasopharynx treated by radiotherapy alone: Determinants of distant metastasis and survival. Radiother. Oncol..

[36] Zhang X., Guo Y., Ye Q., Yang Z., Dong Z. (1999). Study of the relation between MMP2, MMP9 and nasopharyngeal carcinoma. J. Clin. Otolaryngol..

[37] Sun B., Xu M. (2015). Matrine inhibits the migratory and invasive properties of nasopharyngeal carcinoma cells. Mol. Med. Rep..

[38] Ohtsuki T., Yokosawa E., Koyano T., Preeprame S., Kowithayakorn T., Sakai S., Toida T., Ishibashi M. (2008). Quinic acid esters from *Pluchea indica* with collagenase, MMP-2 and MMP-9 inhibitory activities. Phytother. Res..

[39] Safarzadeh E., Shotorbani S.S., Baradaran B. (2014). Herbal medicine as inducers of apoptosis in cancer treatment. Adv. Pharm. Bull..

[40] Hock A.K., Vousden K.H. (2012). Tumor suppression by p53: Fall of the triumvirate?. Cell.

[41] Nayak G., Cooper G.M. (2012). p53 is a major component of the transcriptional and apoptotic program regulated by PI 3-kinase/Akt/GSK3 signaling. Cell Death Differ..

[42] Pflaum J., Schlosser S., Muller M. (2014). p53 family and cellular stress responses in cancer. Front. Oncol..

[43] Beckerman R., Prives C. (2010). Transcriptional regulation by p53. Cold Spring Harb. Perspect. Biol..

[44] Mihara M., Erster S., Zaika A., Petrenko O., Chittenden T., Pancoska P., Moll U.M. (2003). p53 has a direct apoptogenic role at the mitochondria. Mol. Cell.

[45] Elkholi R., Floros K.V., Chipuk J.E. (2011). The role of BH3-only proteins in tumor cell development, signaling, and treatment. Genes Cancer.

[46] Zeng G.-Q., Yi H., Li X.-H., Shi H.-Y., Li C., Li M.-Y., Zhang P.-F., Feng X.-P., Wan X.-X., Qu J.-Q. (2011). Identification of the proteins related to p53-mediated radioresponse in nasopharyngeal carcinoma by proteomic analysis. J. Proteomics.

[47] Pan J., Zhang S., Chen C., Xiao S., Sun Y., Liu C., Su X., Li D., Xu G., Xu B. (2009). Effect of recombinant adenovirus-p53 combined with radiotherapy on long-term prognosis of advanced nasopharyngeal carcinoma. J. Clin. Oncol..

[48] Zapata-Bustos R., Alonso-Castro A.J., Gomez-Sanchez M., Salazar-Olivo L.A. (2014). Ibervillea sonorae (Cucurbitaceae) induces the glucose uptake in human adipocytes by activating a PI3K-independent pathway. J. Ethnopharmacol..

[49] Hwang Y.C., Lu T.Y., Huang D.Y., Kuo Y.S., Kao C.F., Yeh N.H., Wu H.C., Lin C.T. (2009). NOLC1, an enhancer of nasopharyngeal carcinoma progression, is essential for TP53 to regulate MDM2 expression. Am. J. Pathol..

[50] Nakabayashi H., Shimizu K. (2012). Involvement of Akt/NF-kappaB pathway in antitumor effects of parthenolide on glioblastoma cells *in vitro* and *in vivo*. BMC Cancer.

